# Trajectory of IgG to SARS-CoV-2 After Vaccination With BNT162b2 or mRNA-1273 in an Employee Cohort and Comparison With Natural Infection

**DOI:** 10.3389/fimmu.2022.850987

**Published:** 2022-03-21

**Authors:** Behnam Keshavarz, Nathan E. Richards, Lisa J. Workman, Jaimin Patel, Lyndsey M. Muehling, Glenda Canderan, Deborah D. Murphy, Savannah G. Brovero, Samuel M. Ailsworth, Will H. Eschenbacher, Emily C. McGowan, Barbara J. Mann, Michael R. Nelson, Alexandra Kadl, Judith A. Woodfolk, Thomas A.E. Platts-Mills, Jeffrey M. Wilson

**Affiliations:** ^1^ Division of Allergy & Clinical Immunology, Department of Medicine, University of Virginia, Charlottesville, VA, United States; ^2^ Division of Infectious Disease and International Medicine, Department of Medicine, University of Virginia, Charlottesville, VA, United States; ^3^ Division of Pulmonary and Critical Care, Department of Medicine, University of Virginia, Charlottesville, VA, United States; ^4^ Department of Pharmacology, University of Virginia, Charlottesville, VA, United States

**Keywords:** COVID-19, SARS-CoV-2, vaccines, mRNA vaccines, IgG, durability, human coronaviruses (HCoV), OC43

## Abstract

Three COVID-19 vaccines have received FDA-authorization and are in use in the United States, but there is limited head-to-head data on the durability of the immune response elicited by these vaccines. Using a quantitative assay we studied binding IgG antibodies elicited by BNT162b2, mRNA-1273 or Ad26.COV2.S in an employee cohort over a span out to 10 months. Age and sex were explored as response modifiers. Of 234 subjects in the vaccine cohort, 114 received BNT162b2, 114 received mRNA-1273 and six received Ad26.COV2.S. IgG levels measured between seven to 20 days after the second vaccination were similar in recipients of BNT162b2 and mRNA-127 and were ~50-fold higher than in recipients of Ad26.COV2.S. However, by day 21 and at later time points IgG levels elicited by BNT162b2 were lower than mRNA-1273. Accordingly, the IgG decay curve was steeper for BNT162b2 than mRNA-1273. Age was a significant modifier of IgG levels in recipients of BNT162b2, but not mRNA-1273. After six months, IgG levels elicited by BNT162b2, but not mRNA-1273, were lower than IgG levels in patients who had been hospitalized with COVID-19 six months earlier. Similar findings were observed when comparing vaccine-elicited antibodies with steady-state IgG targeting seasonal human coronaviruses. Differential IgG decay could contribute to differences observed in clinical protection over time between BNT162b2 and mRNA-1273.

## Introduction

Since December 2020, two different mRNA-based COVID-19 vaccines have received regulatory authorization for use in the USA and have been used to immunize millions of Americans (1–3). BNT162b2 (Pfizer/BioNTech) and mRNA-1273 (Moderna) each use lipid nanoparticles to deliver mRNA that encodes for a pre-fusion stabilized spike glycoprotein immunogen, and both have shown strong protection in clinical trials and real world experience (4, 5). Nonetheless, there are differences in formulation, mRNA dose, and timing of the prime-boost regimen, factors which could account for differences in clinical protection (6–9). In addition, Ad26.COV2.S (Johnson & Johnson/Janssen) is an adenovirus-based COVID-19 vaccine that has been used in the USA since receiving regulatory approval in late February 2021 (10). In contrast to the prime-boost strategy of the two mRNA vaccines, Ad26.COV.S was introduced to be used as a single immunization. There have been a number of reports that have described the immune response elicited by these different vaccines (4, 5, 11–13). Evidence that antibody levels wane over time is one of the factors that led to the decision by the FDA and CDC to recommend an additional booster shot for select populations (14–17). Despite these recent reports, to date there has been limited head-to-head evaluation of immune responses to these three different COVID-19 vaccines. Here we used a quantitative binding assay to measure IgG to SARS-CoV-2 spike receptor-binding domain (RBD) to evaluate the magnitude and trajectory of antibody responses in an employee vaccine cohort in which all three vaccines were administered. Age and sex were explored as variables that could impact the antibody response. Finally, antibody levels following COVID-19 vaccination were compared to the IgG response generated in patients who were hospitalized with COVID-19 and to steady-state IgG specific for seasonal human coronaviruses (HCoV) in healthy volunteers.

## Materials and Methods

### Study Design and Populations

Adults affiliated with the University of Virginia (UVA) who had or were planning to receive a COVID-19 vaccine were recruited by flyer and UVA health system-wide email announcements between 12/21/2020 and 5/1/2021. Of note, this study expands on a previously reported interim analysis of the same cohort (12). The majority of enrollees in this UVA Institutional Review Board (IRB)-approved study were employed by the UVA Health System. The current analysis included all participants who received a full regimen of BNT162b2 (two inoculations), mRNA-1273 (two inoculations) or Ad26.COV2.S (one inoculation), and had at least one blood sample drawn between 7 and 100 days after the final vaccination in the regimen. No samples were included in this analysis from subjects who received additional COVID-19 vaccinations. Of note, here we used the word “boost” to refer to the second mRNA vaccination. With rare exception, the vaccine that was administered in this convenience sample depended on local vaccine availability. Some subjects provided multiple blood samples, including a sample prior to receiving their first vaccine (baseline sample), at a time point between the first and second mRNA vaccination and a time point >100 days after the final vaccination. A subset of participants had weekly blood draws for 8 weeks following the first vaccination.

Recovered COVID-19 patients who had been previously hospitalized for their disease were recruited from a COVID-19 follow-up clinic (18 **).** In this UVA-approved IRB study, subjects provided written informed consent and blood was drawn at the post-hospital follow-up visit. A subset of these patients also had one or more blood samples available that had been banked during their prior inpatient admission. Initial timing of COVID-19-related symptoms was determined by patient questionnaire during the follow-up visit, and, where available, by chart review of admission medical records. Of these patients, none had received any COVID-19 vaccination at the time of participation.

### Antibody Assays

IgG to SARS-CoV-2 spike RBD and nucleocapsid, and also IgG to HCoV E229 and OC43 spike S1 proteins, were measured with a high-capacity quantitative ImmunoCAP-based assay using a Phadia 250 (Thermo-Fisher/Phadia, Waltham, MA, USA), as previously described (19). In brief, commercially acquired recombinant coronavirus proteins were biotinylated and conjugated to the streptavidin-coated solid phase of the ImmunoCAP. Background was accounted for by subtracting signal to an unconjugated streptavidin ImmunoCAP that was run in tandem with each sample. The cut-off of the assay for IgG to SARS-CoV-2 spike RBD was previously described as 2.5 µg/mL (19). IgG to tetanus toxoid was measured with a commercial ImmunoCAP assay. Of note, the ImmunoCAP assay has an internal heterologous curve control that is used to generate a read-out in µg/mL. SARS-CoV-2 proteins were purchased from RayBiotech (Peachtree Corners, GA) and HCoV spike S1 proteins were purchased from Sino Biological (Wayne, PA, USA). As a validation measure of the binding assay, a subset of 18 samples were evaluated using a SARS-CoV-2 plaque-reduction neutralization assay. The correlation between neutralizing antibody (nAb) titers and IgG levels was moderately strong with a Pearson’s R coefficient of 0.56, p=0.02 (**Supplementary Figure 1** and **Supplementary Methods**).

### Statistical Analysis

Continuous data were compared with Student’s T test, ANOVA, Mann-Whitney U Test or Kruskal-Wallis, as appropriate. Categorical data was compared by Chi squared test. Correlation was assessed with Pearson’s Test. Antibody levels were expressed by geometric mean (GM) with 95% confidence intervals. Regression models were performed on log-transformed data. Subjects with history or serologic evidence (on the basis of a positive nucleocapsid IgG) of prior COVID-19 infection were excluded from secondary analyses involving regression modeling, paired longitudinal data or the exploration of age and sex as modifiers. Statistical analysis was performed with GraphPad Prism 8 (GraphPad Software).

## Results

### Employee Vaccine Cohort

Of 243 participants who were consented and provided a blood sample, 234 received a full vaccine regimen and provided at least one blood sample after the final vaccine. The median age was 41.5, with 34% age 50 or greater, and women represented 74% of the participants (**Table 1**). Age and sex were similarly distributed among participants who received BNT162b2 (n=114), mRNA-1273 (n=114) and Ad26.COV2.S (n=6), with the exception that males were more frequent among recipients of Ad26.COV2.S (67%). Of the 8 participants who reported COVID-19 prior to vaccination, 7 were recipients of BNT162b2 (6%) *vs* only one for mRNA-1273 (1%). An additional 7 subjects had evidence of COVID-19 infection at some point in the study on the basis of personal report or a nucleocapsid IgG level >5 µg/mL, as previously described (12).

**Table 1:** Characteristics of Vaccinated Study Population.

	Total cohort	BNT162b2 (Pfizer/BioNTech)	mRNA-1273 (Moderna)	Ad26.COV2.S (J&J/Janssen)
n	234	114	114	6
Age, median (IQR)	41.5 (32-54)	43.0 (33-56)	39.5 (31-54)	46.5 (36-55)
Age, ≥ 50yrs	79 (34%)	38 (33%)	39 (34%)	2 (33%)
Sex. F	173 (74%)	88 (77%)	83 (73%)	2 (33%)
Sex, M	61 (26%)	26 (23%)	31 (27%)	4 (67%)
Baseline pre-vaccine sample	47 (20%)	23 (20%)	22 (19%)	2 (33%)
Sample between 1st and 2nd vaccine	76 (32%)	38 (33%)	34 (30%)	NA
Sample between 7-100 days after final vaccine	234 (100%)	114 (100%)	114 (100%)	6 (100%)
Sample >100 days after final vaccine	128 (55%)	59 (52%)	63 (55%)	6 (100%)
COVID-19 prior to enrolment*	8 (3%)	7 (6%)	1 (1%)	0 (0%)
e/o COVID-19 during study**	7 (3%)	4 (4%)	3 (3%)	0 (0%)

*Defined by self-report.

**Defined by a nucleocapsid IgG level > 5 µg/mL or self-report.

NA, not available.

### IgG to SARS-CoV-2 Spike RBD Following Vaccination

**Figure 1:**
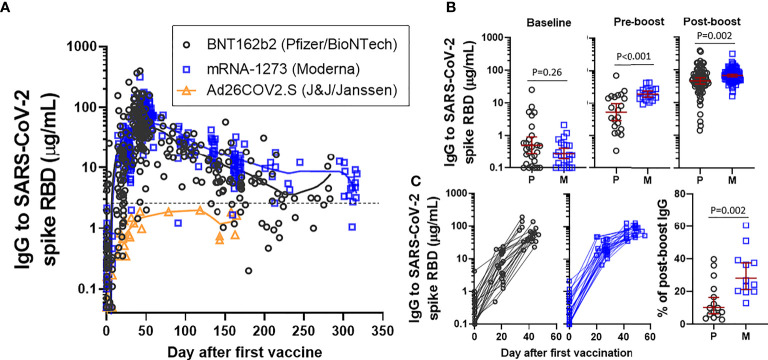
IgG to SARS-CoV-2 spike RBD in an employee cohort. **(A)** Visualization of IgG levels with LOWESS curves in all samples collected from recipients of BNT162b2 (Pfizer/BioNTech), mRNA-1273 (Moderna) or Ad26.COV2.S (J&J/Janssen). The dashed line reflects the cut-off that was originally described for distinguishing positive and negative samples. **(B)** Comparison of levels at baseline, pre-boost (restricted to samples collected within one day of boost) and post-boost (7-31 days after the boost immunization) in recipients of BNT162b2 (P) or mRNA-1273 (M). Presented as GM with 95% CI and comparisons with Mann-Whitney U test. **(C)** Longitudinal data from subjects who had a sample at each of a baseline, pre-boost interval and post-boost interval, excluding subjects with prior COVID-19 infection (left). Level of pre-boost IgG as % in relation to post-boost IgG level, in subjects with longitudinal sampling (right).

LOWESS curve modeling using all available SARS-CoV-2 spike RBD IgG data revealed that responses to BNT162b2 and mRNA-1273 often approached or exceeded 50-100 µg/mL, whereas IgG levels following Ad26.COV2.S did not exceed ~2 µg/mL (**Figure 1A**). We next compared the early immune response elicited by the two mRNA vaccines. SARS-CoV-2 spike RBD IgG levels in samples obtained within one day of the second (boost) vaccination (~3 weeks after BNT162b2 and ~4 weeks after mRNA-1273) were higher in recipients of mRNA-1273 (GM 18.6 µg/mL [95%CI 15-23 µg/mL]) than in recipients of BNT162b2 (GM 5.4 µg/mL [95%CI 3-10 µg/mL]), P<0.001 (**Figure 1B**). Consistent with our previous report, IgG levels from samples collected between 7-31 days following the second immunization were also higher in recipients of mRNA-1273 (GM 68.2 µg/mL [95%CI 62-75 µg/mL]) than in recipients of BNT162b2 (GM 46.9 µg/mL [95%CI 37-59 µg/mL]), P=0.002 (12). The trajectory of the early response was further confirmed by evaluating subjects who had paired longitudinal data. Pre-boost IgG levels as a percentage in relation to the post-boost response were lower in recipients of BNT162b (GM 10.3% [95%CI 6-16%]) than in recipients of mRNA-1273 (GM 28.3% [95%CI 21-38%]), p=0.002 (**Figure 1C**).

**Figure 2:**
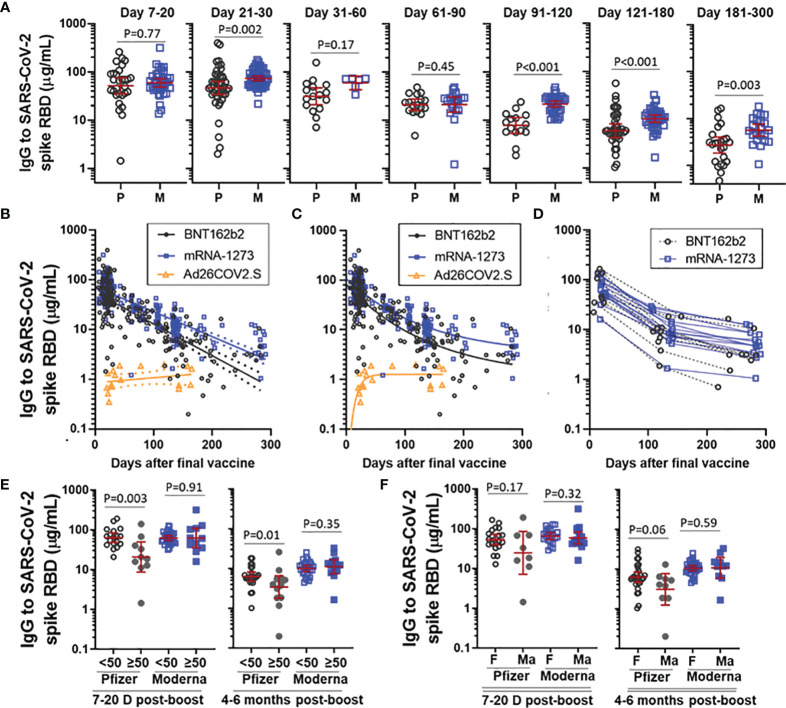
Trajectory of vaccine-elicited IgG over time. **(A)** Comparison of IgG to SARS-CoV-2 spike RBD in recipients of BNT162b2 (P) and mRNA-1273 (M), stratified by the number of days post-boost. Presented as GM with 95% CI and comparisons with Mann-Whitney U test. **(B)** IgG trajectory using linear regression modeling, with dashed lines indicating 95%CI. **(C)** IgG trajectory using one-phase decay model. **(D)** Paired longitudinal data in subjects who had a sample collected at each of an early (Day 7-20), intermediate (Day 100-150) and late (day 200-300) time point. **(E)** IgG to SARS-CoV-2 spike RBD stratified by vaccine and age (<50yrs *vs* ≥50yrs) at D7-20 post-boost and 4-6 months post-boost. **(F)** IgG to SARS-CoV-2 spike RBD stratified by vaccine and sex (female [F] *vs* male [Ma]) at D7-20 post-boost and 4-6 months post-boost. Subjects with prior COVID-19 infection were excluded from analysis in Figs **(B–F)**.

We next assessed antibody decay over time in recipients of the mRNA vaccines. There was no difference in SARS-CoV-2 spike RBD IgG levels between BNT162b and mRNA-1273 at early time points (*ie*, day 7-20) after the second vaccine (**Figure 2A**), however, a difference in IgG levels between BNT162b2 and mRNA-1273 became apparent in samples collected 21-30 days after the second vaccination and the difference was clearly evident from 3 months and onward. Antibody trajectory was further investigated using linear regression. Confidence intervals between BNT162b2 and mRNA-1273 diverged over time and the decay slope for BNT162b2 (-0.0064 [95%CI -0.0072 to -0.0056], R^2 ^= 0.56, P<0.001) was steeper than mRNA-1273 (-0.0050 [95%CI -0.0055 to -0.0045], R^2 ^= 0.69, P<0.001) (**Figure 2B**). A one-phase decay model had relatively strong goodness of fit for both BNT162b2 (R^2 ^= 0.60) and mRNA-1273 (R^2 ^= 0.74) and suggested that the rate of antibody decay slowed down after a relatively rapid early decline (**Figure 2C** **)**. This was supported by evaluating paired longitudinal data from those subjects who had a sample collected at each of three different time intervals after the final vaccine (**Figure 2D**). Recipients of Ad26.COV2.S had relatively stable IgG levels five months out from vaccination in both the linear regression and one-phase decay regression models.

We next explored the effect of age and sex on levels of IgG to SARS-CoV-2 spike RBD elicited by BNT162b2 and mRNA-1273. Building off our earlier work, we stratified subjects by age into a group < 50 years and a group ≥ 50 years (12). Recipients of BNT162b2 who were ≥ 50 years had lower IgG levels than their younger counterparts at both an early post-boost interval (day 7-20) and at 4-6 months (**Figure 2E**). This age difference was not observed for mRNA-1273 at either time point. When stratified by sex, male recipients of BNT162b2, but not male recipients of mRNA-1273, trended toward lower levels than their female counterparts at both the early and later post-boost time interval (**Figure 2F**). Linear regression modeling incorporating age and sex as variables indicated that lower IgG levels were significantly associated with age ≥ 50 years (p=0.04), but not sex (p=0.63), in recipients of BNT162b2, but not mRNA-1273, at day 7-20 post-boost (data not shown).

**Figure 3:**
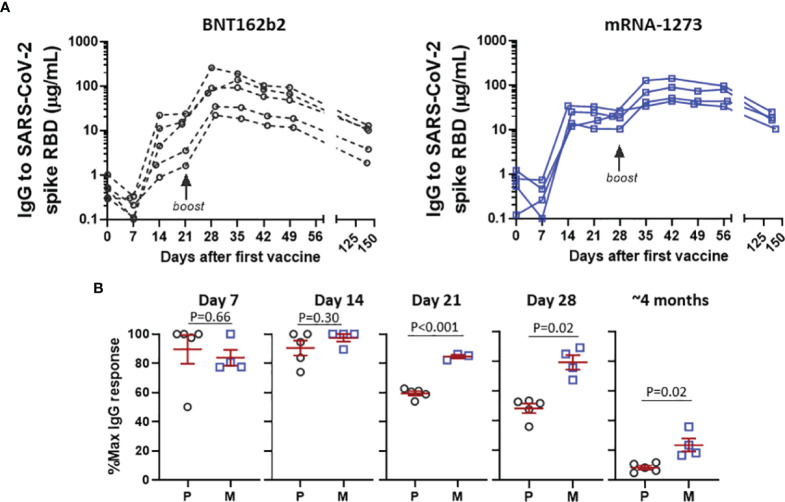
Detailed analysis of response to BNT162b2 P and mRNA-1273 M in subjects with weekly sampling. **(A)** Early IgG response following first and second dose of BNT162b2 or mRNA-1273 in longitudinally monitored subjects. **(B)** Levels of IgG following the second vaccination expressed as % in relation to post-boost peak IgG level, stratified by days after second vaccine. Data in **(B)** presented as GM with 95% CI and comparisons with Mann-Whitney U test.

More detailed analysis of early SARS-CoV-2 spike RBD IgG trajectory was possible for a small subset of the cohort who had samples drawn at weekly intervals for at least 8 weeks following the first mRNA vaccine (**Figure 3A**). Relative levels of IgG elicited by BNT162b2 were similar to the levels elicited by mRNA-1273 at day 7 and day 14 post-boost, however IgG levels dropped substantially (ie, ~40%) as early as 21 days post-boost in recipients of BNT162b2, a decline greater than in recipients of mRNA-1273, P<0.001 (**Figure 3B**). Moreover, the difference in IgG levels between BNT162b2 and mRNA-1273 observed starting at day 21 post-boost persisted over time. Although there were differences in peak levels elicited between individuals, it was notable that antibody decay relative to the peak levels occurred within tight confidence intervals for each vaccine.

### IgG Response After Vaccination *vs*. Severe COVID-19 Infection

**Figure 4:**
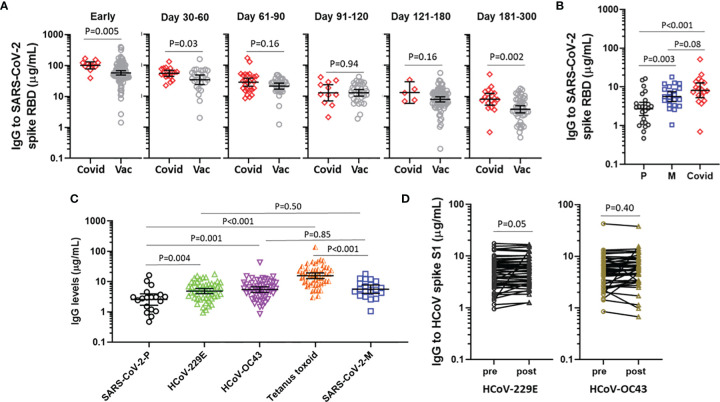
IgG elicited by COVID-19 mRNA vaccines *vs* COVID-19 natural infection and other representative IgG responses. **(A)** IgG levels to SARS-CoV-2 spike RBD following natural infection (Covid) and mRNA-vaccination (Vac), stratified by time in relation to symptom onset or time following the second vaccine. **(B)** IgG levels to SARS-CoV-2 spike RBD at 180-300 days following BNT162b2 (P), mRNA-1273 (M) or natural infection (Covid). **(C)** IgG to SARS-CoV-2 spike RBD ≥6 months following BNT162b2 (P) (n=19) or mRNA-1273 (M) (n=20), compared with IgG to seasonal coronaviruses and tetanus toxoid in 49 representative subjects. Data presented as GM with 95% CI and comparisons with Mann-Whitney U test. **(D)** IgG to HCoV spike S1 in 49 subjects sampled pre-vaccine and post-boost. Paired log transformed data was compared with the Student’s paired T Test.

We next sought to compare IgG levels elicited by vaccination with the levels occurring as a consequence of natural infection in 65 patients evaluated in a post-COVID-19 recovery clinic, none of whom had received a SARS-CoV-2 vaccine. Notably, the COVID-19 patients were older (median age 52.0 [IQR 45-65]) and had a greater proportion of males (60%) than the vaccine cohort, with many patients meeting criteria for severe or very severe COVID-19 on the basis of ICU admission and mechanical ventilation (**Supplementary Table 1**). Among 11 patients who had a sample available at day 7 of their hospital admission (median 17 days post-symptom onset [IQR 14-21]), the IgG levels exceeded the levels observed in samples collected from vaccines between 10-21 days after receipt of two doses of an mRNA vaccine, p=0.005 (**Figure 4A**). Samples collected from patients and vaccines between 2 and 6 months had similar levels, but after 6 months levels were again higher in the COVID-19 patients. When vaccines were stratified by vaccine received, recipients of BNT162b2, but not mRNA-1273, had significantly lower IgG levels than COVID-19 patients after 6 months (**Figure 4B**).

### Vaccine-Elicited IgG Response to SARS-CoV-2 as Compared to IgG to HCoV and Tetanus Toxoid

Finally, we sought to contextualize the levels of IgG that were elicited by COVID-19 vaccination with the IgG levels elicited by an unrelated vaccine that is widely used to immunize adults (i.e., tetanus toxoid vaccine) and also steady-state IgG to representative HCoV. Reference levels for IgG to HCoV and tetanus toxin were established by investigating 49 vaccine recipients who had a blood sample available prior to the first vaccine. Among recipients of BNT162b2 who had samples collected between 6 and 10 months, levels of IgG to SARS-CoV-2 spike RBD were lower than IgG levels to each of HCoV-229E S1, HCoV-OC43 S1 and tetanus toxoid.By contrast, among recipients of mRNA-1273 who had samples collected between 6 and 10 months the IgG levels were similar to IgG to HCoV-229E S1 and HCoV-OC43 S1, but lower than IgG to (**Figure 4C**). Of note, the IgG response to HCoV were similar whether measured at baseline or at the post-boost time interval (**Figure 4D**), speaking to the specificity of the respective assays. We did not observe any association between the IgG response to HCoV spike and the IgG response to SARS-CoV-2 spike RBD (data not shown).

## Discussion

The current findings indicate that BNT162b2 and mRNA-1273 both achieve similar peak IgG levels to the SARS-CoV-2 spike RBD antigen following two vaccine doses. Although this finding is at odds with some earlier reports (11, 20, 21), including an interim analysis of the current cohort (12), it is at least partially explained by different rates of antibody decay that occur following immunization with BNT162b2 and mRNA-1273 and is supported by a recent report from Montoya et al (22). The detailed kinetic analysis here reveals that IgG levels in recipients of BNT162b2 are dropping markedly (eg, 40%), and to a greater extent than mRNA-1273, as soon as 21 days after the booster immunization. Thus, blood samples that are collected over a time frame that extends out past three weeks post-boost, particularly in the case of BNT162b2, are likely not capturing peak IgG levels. Although there is no specific IgG value that is known to be a correlate of immune protection, observed differences in antibody induction and decay suggests that mRNA-1273 could promote more durable humoral immunity than BNT162b2. Of note, there have been reports indicating that breakthrough COVID-19 infections following immunization are fewer in recipients of mRNA-1273 than BNT162b2 (6–8).

Consistent with an interim analysis of this cohort and some other reports, here we observed that relatively older recipients of BNT162b2 had a diminished IgG response as compared to younger vaccine recipients (12, 14, 23–26). This was observed in samples collected in the first three weeks and also confirmed at 4-6 months post-boost. There was no such difference based on age stratification in recipients of mRNA-1273. We also explored sex as a modifier of the vaccine response. There was a trend toward a diminished response in male versus female recipients of BNT162b2, but unlike a prior report, this was not statistically significant (25).

The durability of immune protection following vaccination as compared to natural infection remains an important question (27). Comparing IgG levels at early time points and over time we observed general similarities in the trajectory of IgG decline in recipients of mRNA vaccines and in patients who had been hospitalized with COVID-19. This information could be relevant to public health measures to control the pandemic, though it is important to emphasize that we studied patients with severe COVID-19 whereas the majority of cases in the population are thought to be mild or asymptomatic and to have lower IgG levels (28).

Owing to local immunization patterns, the number of subjects who received Ad26.COV2.S was limited to six. This small sample size precluded detailed studies of kinetics or investigation of age or sex as effect modifiers. Nonetheless, the data revealed that peak IgG levels induced by Ad26.COV2.S were on the order of one to two log orders lower than the levels elicited by the mRNA vaccines. Generally this fits with recent reports that have compared antibody responses in recipients of BNT162b2 with Ad26.COV2.S (29–31). On the other hand, IgG levels appear to be more durable following Ad26.COV2, with levels in some subjects changing little between the first and fifth month after immunization.

While SARS-CoV-2 is the cause of the current COVID-19 pandemic, there are several other coronaviruses that act as human pathogens and contribute to seasonal respiratory infections. Two such coronaviruses (HCoV) strains that circulate widely in the population are E229, an α-coronavirus, and OC43, a β-coronavirus. Natural infection with these HCoV is known to lead to IgG antibodies against the relevant spike proteins, with IgG levels thought to be maintained because of frequent re-infection (32). Here we found that 6 months after immunization with BNT162b2 that IgG levels against SARS-CoV-2 spike RBD were lower than the steady-state levels of IgG to E229 and OC43 spike. Given that steady-state IgG levels to HCoV are thought to be inadequate to prevent HCoV re-infection, it is perhaps not surprising that when IgG levels to SARS-CoV-2 drop to similar or lower levels that rates of breakthrough COVID-19 increase (33).

There are also other limitations to consider. Our assay utilized a recombinant spike RBD based on the original SARS-CoV-2 strain. Thus, antibody binding avidity to recently emerged variants such as delta or omicron could be lower than values we report here. On the other hand, there is no IgG cut-off that has been shown to be a correlate of protection. We did not systematically assess post-vaccination COVID-19 infections, limiting our ability to assess clinical outcomes. Neutralization assays were only carried out on a limited number of samples and we did not investigate B or T cell responses in any of the samples. On the other hand, we have shown that our quantitative binding assay had moderately strong correlation with neutralizing antibodies. In addition, binding assays have several advantages over neutralizing assays when it comes to cost, reproducibility, accuracy and ease of population-based implementation.

In summary, using a quantitative assay to measure IgG that is specific for SARS-CoV-2 spike RBD we have shown that rates of decay are generally similar following mRNA-based COVID-19 vaccination and following natural infection in patients who had severe COVID-19. However, comparison of the IgG response between the two FDA-approved COVID-19 mRNA vaccines revealed that IgG elicited by BNT162b2 rises more slowly, decays more rapidly and the levels are more likely to be impacted by older age and male sex, as compared to IgG elicited by mRNA-1273. Whether these observations are explained by differences between BNT162b2 and mRNA-1273 dose (*ie*, 30 µg *vs* 100 µg), timing of the prime-boost regimen (*ie*, 3 weeks *vs* 4 weeks), or other factors remains to be determined. While it remains an open question whether the differences in IgG levels between BNT162b2 and mRNA-1273 translate to differences in clinical protection, a direct protective role for IgG against COVID-19 is supported by the efficacy of passive immunization with anti-spike monoclonal antibodies (34, 35).

## Data Availability Statement

The raw data supporting the conclusions of this article will be made available by the authors, without undue reservation.

## Ethics Statement

The studies involving human participants were reviewed and approved by University of Virginia Health System Institutional Review Board. The patients/participants provided their written informed consent to participate in this study.

## Author Contributions

BK is first author and JMW was the principal investigator of the study. BK, NR, LJW, MN, TP-M, and JMW conceived the study design. NR, LW, and JMW developed the study protocol. BK and JMW verified the underlying data. All authors contributed to acquisition, analysis or interpretation of data. BK, LW, TP-M, and JMW developed the COVID-19 IgG assay and BK carried out antibody assays. BK and JMW wrote the first draft of the manuscript and all authors provided critical feedback on the paper. All authors had full access to the data and accept responsibility for submission. All authors contributed to the article and approved the submitted version.

## Funding

JMW had salary support from the AAAAI Faculty Development Award and funding for this project from the UVA Manning COVID-19 Research Fund. TP-M receives support from NIH R37-AI20565 which supported the study. The post-COVID cohort recruitment was supported and funded by NIH R21-AI160334 and the University of Virginia School of Medicine GAP Award to JAW. Funding sources had no role in study design, data acquisition, analysis, manuscript preparation, or decision to submit for publication.

## Conflict of Interest

TP-M and JMW have received assay support from Thermo-Fisher/Phadia, but not for work related to this project. JMW has received consultancy fees from Thermo-Fisher/Phadia for work unrelated to this project.

The remaining authors declare that the research was conducted in the absence of any commercial or financial relationships that could be construed as a potential conflict of interest.

## Publisher’s Note

All claims expressed in this article are solely those of the authors and do not necessarily represent those of their affiliated organizations, or those of the publisher, the editors and the reviewers. Any product that may be evaluated in this article, or claim that may be made by its manufacturer, is not guaranteed or endorsed by the publisher.
