# Supplementary material for: Trajectory of IgG to SARS-CoV-2 After Vaccination With BNT162b2 or mRNA-1273 in an Employee Cohort and Comparison With Natural Infection
Source: Front Immunol. 2022 Mar 21;13:850987. doi: 10.3389/fimmu.2022.850987 (PMC8978955; doi:10.3389/fimmu.2022.850987)
Supplement: Supplementary file 1 [file DataSheet_1.docx]

Supplementary Material

# Supplementary Methods

**SARS-CoV-2 Plaque Reduction Neutralization Test (PRNT)**

2.5 x 10^5^ Vero E6 cells were plated onto 12-well plates and incubated overnight. Serum samples, which had been heat-inactivated at 56°C for 30 minutes prior to use, were serially-diluted at 1:40, 1:200, 1:1000 and 1:5000 before mixing 1:1 with SARS-CoV-2 virus, (Hong Kong/VM20001061/2020, NR-52282, BEI Resources) and incubating for 1 hr. The serum-virus solution was then added to the plated Vero E6 cells and incubated for 2 hrs. An Avicel solution containing DMEM with 2.5% FBS and 1.2% Avicel was then layered on top of the cells and incubated for two days at 37˚C/5% CO2. The plate was then fixed with 10% formaldehyde and stained with crystal violet. Plaques were visually enumerated. The antibody dilution that resulted in a 50% reduction in plaques compared to the control was considered the PRNT_50_.

# Supplementary Table

**Supplementary Table 1.** Characteristics of COVID-19 infection cohort and comparison with employee vaccine cohort

| **Characteristic** | **Hospitalized COVID-19 patients seen in follow-up clinic**  **(n=65)** | **Employee vaccine cohort**  **(n=234)** | **p** |
| --- | --- | --- | --- |
| Age, median (IQR) | 52.0 (45-65) | 41.5 (32-54) | <0.001 |
| Age, ≥ 50 yrs, n (%) | 42 (65%) | 79 (34%) | <0.001 |
| Sex, female | 36 (40%) | 173 (74%) | <0.001 |
| Sex, male | 39 (60%) | 61 (26%) | <0.001 |
| Admitted to ICU | 60 (92%) | na | na |
| Mechanical ventilation | 47 (72%) | na | na |
| Sample available from inpatient admission | 11 (17%) | na | na |

## Supplementary Figure


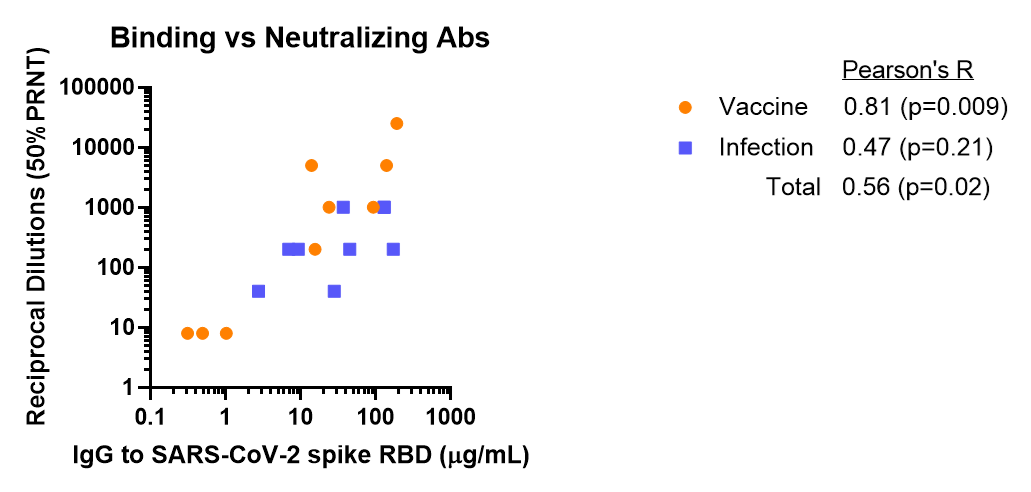


**Supplementary Figure 1.** **IgG to SARS-CoV-2 spike RBD in relation to Plaque-Reduction Neutralization Test (PRNT).** Nine samples from the vaccine cohort and nine samples from patients recovering from COVID-19 infection were assessed by PRNT, defined as 50% reciprocal titers.
